# Hook-up of GluA2, GRIP and liprin-α for cholinergic muscarinic receptor-dependent LTD in the hippocampus

**DOI:** 10.1186/1756-6606-2-17

**Published:** 2009-06-17

**Authors:** Long-Jun Wu, Yu-Tian Wang, Min Zhuo

**Affiliations:** 1 Department of Physiology, Faculty of Medicine, University of Toronto, 1 King's College Circle, Toronto, Ontario M5S 1A8, Canada; 2 Brain Research Centre, University of British Columbia, Vancouver, V6T 1Z3, Canada; 3 Department of Brain and Cognitive Sciences, Seoul National University, Seoul 151-746, Korea

## Abstract

The molecular mechanism underlying muscarinic acetylcholine receptor-dependent LTD (mAChR-LTD) in the hippocampus is less studied. In a recent study, a novel mechanism is described. The induction of mAChR-LTD required the activation of protein tyrosine phosphatase (PTP), and the expression was mediated by AMPA receptor endocytosis via interactions between GluA2, GRIP and liprin-α. The hook-up of these proteins may result in the recruitment of leukocyte common antigen-related receptor (LAR), a PTP that is known to be involved in AMPA receptor trafficking. Interestingly, the similar molecular interaction cannot be applied to mGluR-LTD, despite the fact that the same G-protein involved in LTD is activated by both mAChR and mGluR. This discovery provides key molecular insights for cholinergic dependent cognitive function, and mAChR-LTD can serve as a useful cellular model for studying the roles of cholinergic mechanism in learning and memory.

## Editorial

Activity-dependent changes in synaptic strength, such as long-term potentiation (LTP) and long-term depression (LTD), are thought to be the cellular models of learning and memory [1]. In the hippocampus, several mechanistically distinct forms of LTD have been reported. Two main forms of LTD (NMDA receptor (NMDAR)- and metabotropic glutamate receptor (mGluR)-dependent LTD), have been intensively studied at hippocampal CA1 region and the signaling pathways underlying LTD have been established. For example, NMDAR-LTD can be induced by prolonged low frequency stimulation (LFS). The mechanisms underlying NMDAR-LTD include activation of NMDA receptor, postsynaptic Ca^2+ ^elevation, and subsequent activation of protein phosphatases. The AMPA receptor endocytosis is likely important for the expression of the depression. mGluR-LTD can be induced following paired-pulse LFS or DHPG (Group I mGluR agonist) application, and is dependent on the activation of group I mGluR. Similar postsynaptic AMPA receptor endocytosis and possible presynaptic reduction of glutamate release are thought to contribute to reduced responses [2-6].

Less attention has been paid to another form of LTD, one which is dependent on muscarinic acetylcholine receptors (mAChR). First described in the visual cortex, mAChR-LTD was subsequently found in various brain regions, including the perirhinal cortex and hippocampus [7-11]. Cholinergic neurotransmission has been long implicated in memory and cognition [12] and thus mAChR-LTD is proposed to be critical for cholinergic-related brain functions and disease. However, the molecular mechanisms underlying mAChR-LTD remain largely unknown. Since mAChRs and mGluRs activate similar signaling pathways involving the same G-proteins and isoforms of phospholipase C (PLC) [13], it is reasonable to expect that mAChR-LTD and mGluR-LTD share similar molecular mechanisms. However, a recent study by Dickinson and colleagues [this issue, Dickinson et al., Molecular Brain] sheds new light on the molecular mechanisms underlying mAChR-LTD in the hippocampus. Using whole-cell patch clamp recording in hippocampal CA1 pyramidal neurons from 4–5 week-old young rats, they found that mAChR-LTD requires the activation of M1 receptors and protein tyrosine phosphatases (PTPs), which in turn result in AMPA receptor internalization via interactions between GluA2, GRIP and liprin-α. However, these same molecular interactions are not required for mGluR-LTD in the hippocampus.

The major cholinergic innervations of the hippocampus come from the medial septum [14]. Five different muscarinic receptor subtypes (M1-5) have been identified, all of them are expressed in the hippocampus. Among them, M1, 3, 5 are coupled to the Gq/11 and phospholipase C (PLC) pathway, while M2 and 4 receptors negatively regulate adenylyl cyclases [15]. In the hippocampal CA1 region, mAChR-LTD has only recently been identified. It is induced following application of the cholinergic muscarinic receptor agonist, carbachol (CCh) to hippocampal slices [7,11]. Here Cho and colleagues demonstrate that mAChR-LTD does not require the involvement of NMDA receptors. Moreover, mAChR-LTD occurs independently of mGluRs. These findings support the argument that mAchR-LTD represents a unique form of LTD within the hippocampus. Consistent with previous studies, M1 receptors were found to contribute to the induction of mAChR-LTD. This is demonstrated by the use of a selective M1 receptor agonist 77-LH-28-1, and the antagonist, pirenzepine. The cholinergic M1 receptor is known to link to Gq and the subsequent PLC signaling pathway, which induces Ca^2+ ^release from intracellular Ca^2+ ^store and activation of PKC. To further test whether mAChR-LTD is Ca^2+^- or PKC-dependent, cyclopiazonic acid (for Ca^2+ ^store depletion), BAPTA (Ca^2+ ^chelator), Ro 32-0432 (PKC inhibitor) and PKC19-31 (inhibitory peptide for PKC) were used and none was shown to affect mAChR-LTD, providing the strong evidence that mAchR-LTD may employ special signaling pathways. Postsynaptic application of GDPβ S, a G-protein inhibitor, abolished mAChR-LTD. Taken together, these results suggest that the induction of mAChR-LTD is dependent on M1 receptors and a G-protein signaling mechanism, but not on a conventional Gq-coupled pathway.

Protein phosphatases are known to contribute to hippocampal LTD [6]. For example, serine/threonine protein phosphatases PP1 and PP2B are required for NMDAR-LTD, while protein tyrosine phosphatase (PTP) is required for mGluR-LTD [6,16,17]. Therefore, Dickinson and colleagues set out to study whether these phosphatases play roles in mAChR-LTD. By postsynaptic application of phosphatase inhibitors, they found that PTP, but not serine/threonine protein phosphatases, is required for mAChR-LTD. In addition, they also found that protein synthesis is not required for mAChR-LTD. This result is quite surprising, since protein synthesis has been found to be required for both mGluR-LTD and mAChR-LTD in other studies [7,10,18]. It has been reported that extracellular signal-regulated kinase (ERK) and mammalian target of rapamycin (mTOR) translational activation pathways contribute to protein synthesis-dependent LTD [7,19]. Because different results have been reported in term of the requirement of protein synthesis in LTD, it would worthwhile in future studies to examine the roles of ERK and mTOR pathways in mAChR-LTD.

How then is mAChR-LTD expressed? AMPA receptor endocytosis is the key expression mechanism for both NMDAR-LTD and mGluR-LTD [3,6,20,21]. In a previous report, the surface GluA1 internalization has been observed following CCh treatment in cultured hippocampal neurons [7]. To examine the expression mechanism of mAChR-LTD, Dickinson et al. compared the cell surface and total expression level of GluA2 in control versus CCh-treated hippocampal slices. Following CCh treatment they observed a reduction in GluA2 expression on the cell surface, while total expression levels of GluA2 remained largely unchanged. These results suggest a significant increase in GluA2-containing AMPA receptor endocytosis following LTD induction.

A number of studies have revealed that AMPA receptor-interacting proteins such as NSF, AP2, GRIP, ABP, and PICK1 are critically involved in AMPA receptor trafficking related synaptic plasticity [3,20,22]. Most notably, GRIP/ABP and/or PICK1 are required for NMDAR-LTD in hippocampus, cerebellum and cortex, while PICK1 is required for mGluR-LTD in ventral tegmental area, cerebellum and perirhinal cortex [3,22]. However, it is still unknown which interacting proteins are involved in mAChR-LTD. To address this question, Dickinson et al. used peptide inhibitors to block either the interaction between GluA2 and PICK1 (pep2-EVKI), or between GluA2 and GRIP/ABP as well as PICK1 (pep2-SVKI). They found that pep2-SVKI, but not pep2-EVKI, inhibited mAChR-LTD, suggesting that GRIP rather than PICK1 is involved in mAChR-LTD. Interestingly, pep2-SVKI did not block mGluR-LTD, which suggests that neither GRIP nor PICK1 is involved. These findings indicate a different mechanism is likely involved at the level of AMPA receptor trafficking between mGluR- and mAChR-LTD.

To further examine the mechanisms by which GRIP modulates GluA2 trafficking and mAChR-LTD, they focused on the GRIP interacting protein, Liprin-α, which can directly interact with GRIP via its PDZ6 domain [23]. They found that disrupting the interaction between GRIP and liprin-α using a synthetic peptide selectively blocked mAChR-LTD but not mGluR-LTD and NMDAR-LTD. These results suggest that the GRIP-liprin-α interaction is specifically required for mAChR-LTD. Liprin-α could recruit leukocyte common antigen-related receptors (LAR), a PTP known to be involved in AMPA receptor trafficking, axon guidance and neuronal development [24]. One attractive hypothesis is that activation of LAR phosphatase is triggered in mAChR-LTD via it's interaction with the liprin-α-GRIP-GluA2 complex and the subsequent tyrosine dephosphorylation of GluA2. GluA2 tyrosine dephosphorylation results in the release of GluA2 from GRIP and AMPA receptor endocytosis, thereby expressing LTD. Indeed, in the present study, spectrum PTP inhibitors which can inhibit LAR phosphatase activity are effective in blocking mAChR-LTD. In addition, it has been reported that disruption of GRIP-liprin interactions, or knockdown of LAR interfere with dendritic AMPA receptor distribution [23,24].

In summary, Dickinson et al. examined the detailed induction and expression mechanisms of mAChR-LTD in hippocampal CA1 region. They nicely demonstrated that cholinergic M1 receptors, G-protein signaling, PTP activity and GluA2 internalization are involved in mAChR-LTD. More importantly, their results reveal that the interaction of GluA2-GRIP-liprin-α is required for mAChR-LTD, the mechanisms of which may require the recruitment of LAR, GluA2 tyrosine dephosphorylation, and thus AMPA receptor endocytosis. The present study unveils a novel cellular mechanism for mAChR-LTD, which is different from mGluR-LTD. Future studies are needed to address how the activation of M1 receptor leads to the recruitment of LAR-liprin-α-GRIP-GluA2 pathway. In addition, gene knockout mice deficient of AMPA receptor subunits can be used to evaluate/confirm the roles of these subunits in mAChR-LTD. Lastly, it would be valuable to identify whether the mechanisms observed in the present study are required for synaptically-induced mAChR-LTD in vivo and the pathophysiological relevance of heterosynaptic mAChR-LTD in brains under disease conditions.
